# Internal hernia caused by bridge formation between the medial and lateral segments of the liver: a case report

**DOI:** 10.1186/s12876-022-02294-x

**Published:** 2022-06-03

**Authors:** Kohei Kanamori, Takashi Ogimi, Lin Fung Chan, Hiroshi Miyakita, Kazutake Okada, Hajime Kayano, Masaki Mori, Toshio Nakagohri, Kazuo Koyanagi, Seiichiro Yamamoto

**Affiliations:** Department of Gastroenterological Surgery, Tokai University School of Medicine, 143 Shimokasuya, Isehara, Kanagawa 259-1193 Japan

**Keywords:** Hepatic bridge, Internal hernia, Small bowel obstruction, laparoscopic surgery, Case report

## Abstract

**Background:**

Despite numerous reports on ischemic bowel obstruction caused by internal hernia, no case presentation has been reported of an internal hernia caused by a bridge formed between the medial and lateral zones of the liver. Herein, we report the first case of ischemic bowel obstruction caused by a hepatic bridge.

**Case presentation:**

A 24-year-old man complaining of abdominal pain was referred to our hospital and admitted. Computed tomography showed formation of a closed loop of small bowel with a hernia orifice near the hilar region, and poor contrast of the prolapsed small bowel. We suspected ischemic bowel obstruction caused by an internal hernia with a fissure of the greater omentum as the hernia orifice, and performed emergency surgery. Laparoscopic observation revealed that the medial and lateral segments of the liver formed a bridge on the dorsal side at the liver portal, and that the small intestine was ischemic in the gap created between the bridge and the medial and lateral liver segments. A Meckel’s diverticulum was also invaginated in the gap. The bridge was dissected out and the hernia orifice was opened to release the bowel obstruction. The small bowel was preserved and the Meckel’s diverticulum was resected. The patient’s postoperative course was uneventful.

**Conclusions:**

We experienced a case of ischemic bowel obstruction caused by hepatic bridge formation, which was successfully treated by laparoscopic surgery.

**Supplementary Information:**

The online version contains supplementary material available at 10.1186/s12876-022-02294-x.

## Background

An internal hernia is defined as the protrusion of abdominal viscera, most commonly small bowel loops, through a peritoneal or mesenteric aperture into a compartment in the abdominal and pelvic cavity [1, 2]. Internal hernia is diagnosed by characteristic findings on computed tomography (CT) imaging involving a closed-loop formation, although the utility of CT imaging for identifying the hernia orifice is limited [3].

Steinke classified internal hernias into two types: fossa hernias, in which organs are inserted into the peritoneal fossa or scrotum of the abdominal cavity, and hiatal hernias, in which organs are inserted into an abnormal hiatus [4]. The gap in the mesentery after anastomosis of the gastrointestinal tract can also provide an entrance to a hernia, as first reported by Petersen in 1900 [5]. Despite the numerous reports of intestinal obstruction related to these etiologies, there are no reports of a ischemic intestinal obstruction due to formation of a bridge between the parenchyma of the liver.

Herein, we present a novel case of an extremely rare type of primary visceral hernia that presented with ischemia of the small bowel and invagination of a Meckel’s diverticulum caused by formation of a bridge between the medial and lateral segments of the liver.

## Case presentation

**Fig. 1:**
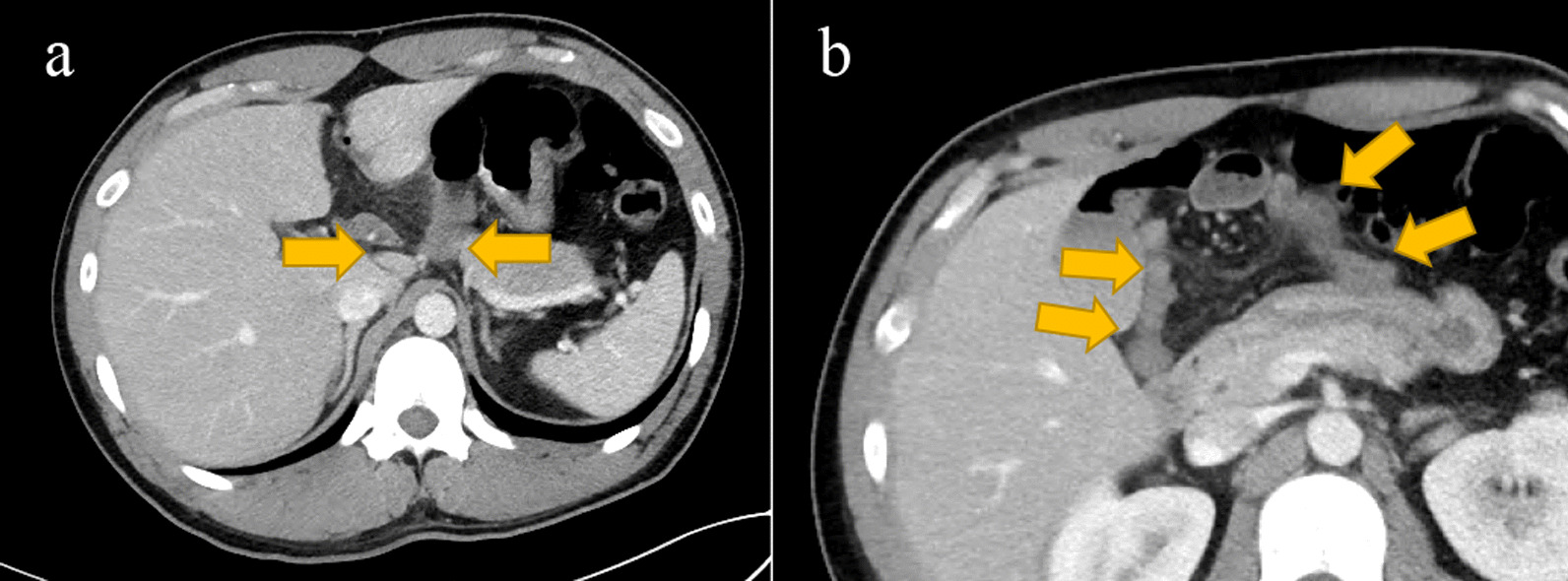
Contrast-enhanced computed tomography (CT) imaging. **a** The formation of a closed loop with a hernia portal near the hilar region. **b** Poor contrast of the prolapsed small intestine

A 24-year-old man with comorbidities of hypertension and atopic dermatitis and no history of abdominal surgery presented to the previous hospital with a chief complaint of upper abdominal pain. Blood tests and CT scan showed no abnormalities. Afterwards, his abdominal pain worsened and he visited our emergency room. He had localized tenderness in the upper abdomen but no signs of peritoneal irritation. The patient was admitted to hospital for follow-up because of severe abdominal pain. The next morning, a further increase in abdominal pain was observed. CT showed the formation of a closed loop of the small bowel with a hernia orifice near the hilar region and poor contrast of the prolapsed small bowel (Fig. 1). We suspected ischemic bowel obstruction caused by an internal hernia with a fissure of the greater omentum as the hernia orifice, and performed emergency surgery.

**Fig. 2:**
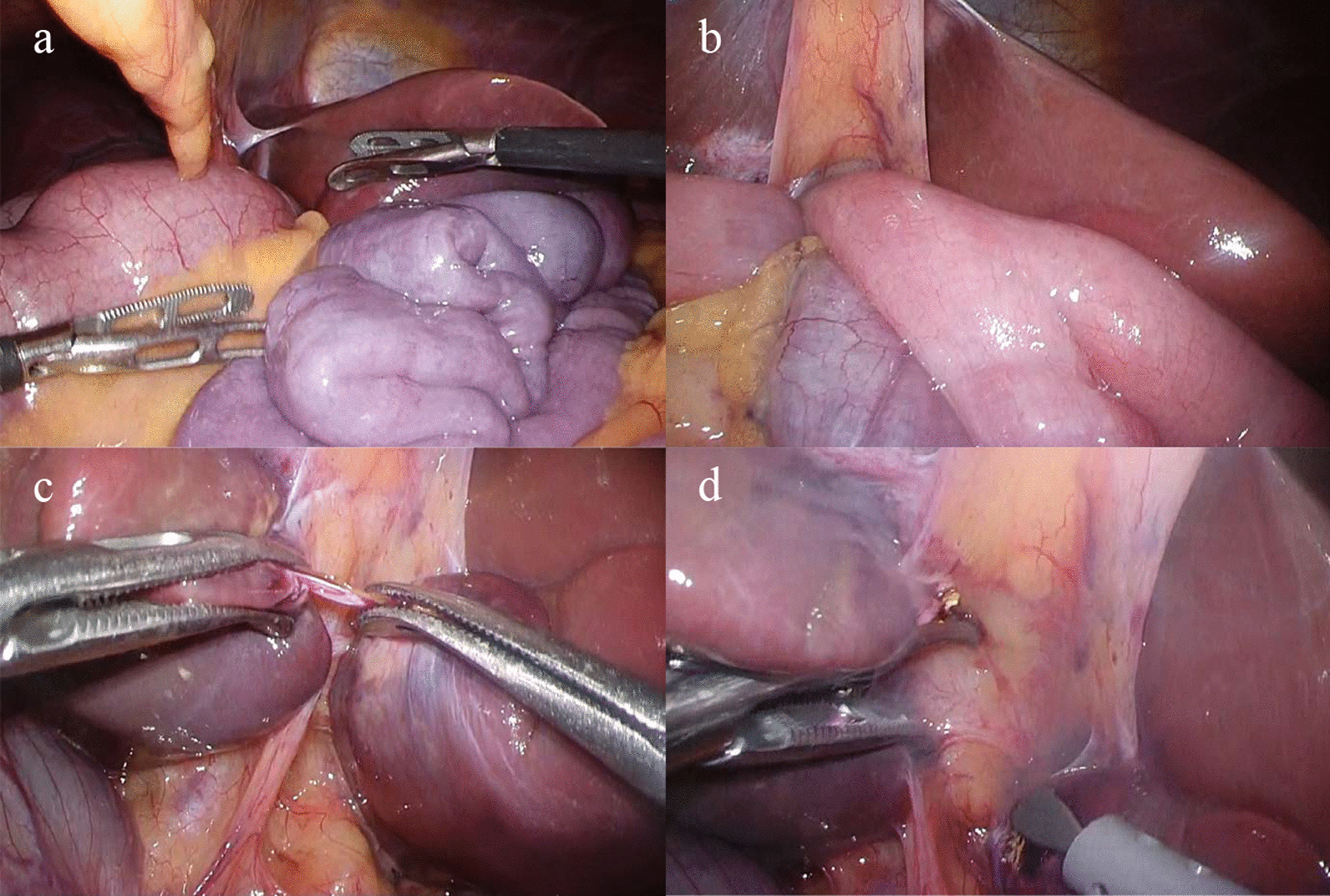
Intraoperative findings. **a** Small intestine with poor coloration caused by an inclusion. **b** Small intestine inserted into the hernia portal. **c** The hernia portal between the hepatic bridge and the hepatic condyles. **d** The hernia portal after release

**Fig. 3:**
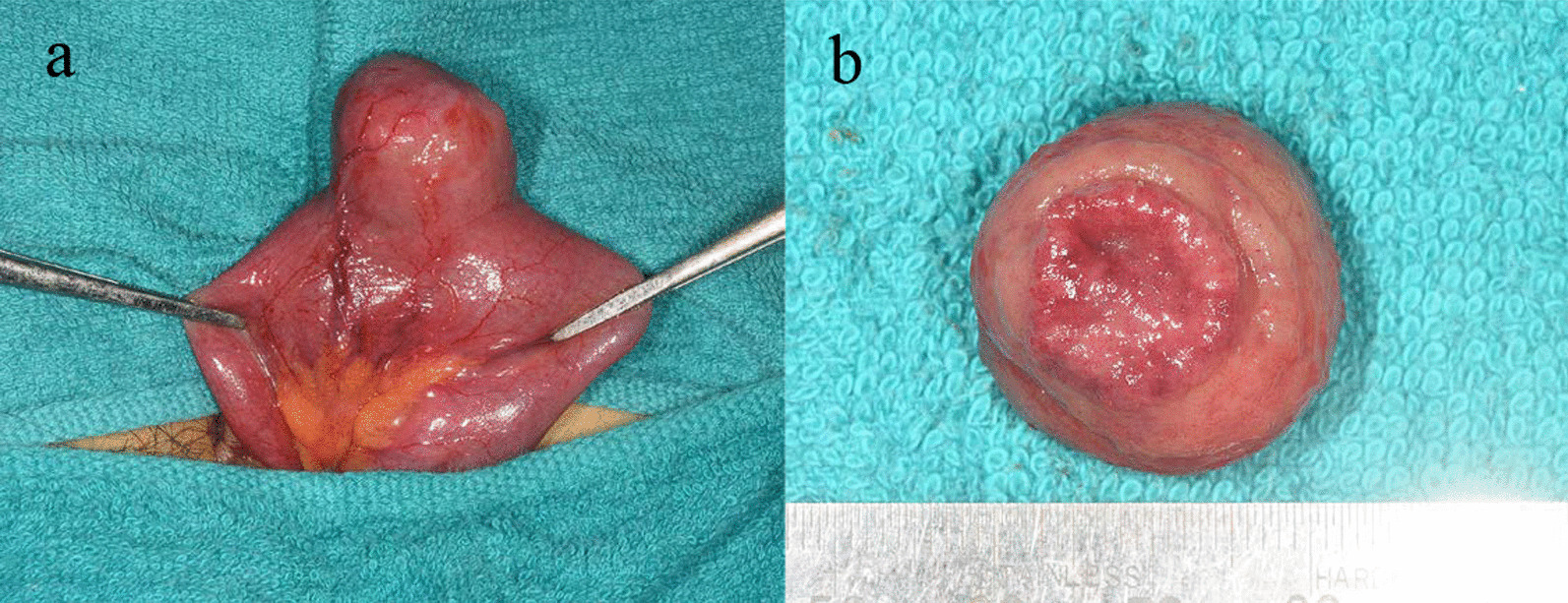
Meckel’s diverticula. **a** Meckel’s diverticulum pulled out of the abdomen through a small laparotomy wound. **b** The resected specimen showed ectopic gastric mucosa

Laparoscopic observation revealed that the medial and lateral segments of the liver formed a bridge on the ventral side at the liver portal, and the small bowel was ischemic in the gap created between the bridge and medial and lateral liver segments (Fig. 2). A Meckel’s diverticulum was also found in the same area. The bridge was dissected out and the hernia portal was opened to release the bowel obstruction. The small bowel was slightly pale, but it improved and was preserved after the ischemia was released. The Meckel’s diverticulum was resected under a small laparotomy with the intraoperative consent of the patient’s father, a physician (Fig. 3). This procedure is shown in more detail in an additional movie file (see Additional file 1).

The patient had an uneventful postoperative course and was discharged on the fourth postoperative day. Histopathological examination of the Meckel’s diverticulum showed ectopic gastric mucosa without malignancy.

## Discussion and conclusions

We experienced a previously unreported type of small bowel obstruction caused by an internal hernia. Although preoperative imaging revealed findings characteristic of an internal hernia, we were unable to differentiate it from a hiatal hernia because of the anatomical similarity of the hernia orifice.

Bridges between the lateral and medial segments of the liver are often experienced in routine surgery. In an intraoperative search of 104 patients, Sugarbaker et al. reported that 49% had a bridge between the parenchyma of the liver (i.e., a ‘hepatic bridge’) [6]. The gap between the hepatic bridge and the hepatic conduit is usually narrow enough that the intestinal tract cannot pass through it. To our knowledge, there are no previous reports of an internal hernia through this gap. In the present case, the bridge was so thin that it was forced apart by the inserted small bowel at the time of surgery, resulting in a large gap.

Laparoscopic surgery for ischemic bowel obstruction was reported to be safe in patients with a stable general condition and a mild intestinal dilatation, which allows for a clear view of the abdominal cavity [7]. Thus, it is acceptable to perform this procedure while considering the potential for conversion to laparotomy. In the present case, the bowel obstruction was safely released laparoscopically, while a small laparotomy was required later for resection of the Meckel’s diverticulum.

The Meckel’s diverticulum was found incidentally in our patient during surgery, but was not considered to be a direct cause of the internal hernia. Most Meckel’s diverticula are asymptomatic, with only 6.4% of patients developing symptoms [8]. Although the decision to excise an asymptomatic Meckel’s diverticulum is controversial, we decided to resect it with the intraoperative consent of the patient’s father, a physician.

In summary, we experienced a case of ischemic bowel obstruction caused by hepatic bridge formation, which was successfully treated by laparoscopic surgery.

## Supplementary Information

**Additional file 1**. Video of laparoscopic surgery. The data is an edited video of the laparoscopic surgery, including the intra-abdominal view, release of the internal hernia, and opening of the hernia orifice.

## Data Availability

All data generated during this study are included in this published article.

## Abbreviation

CT: Computed tomography
